# Modeling Photodissociation: Quantum Dynamics Simulations of Methanol

**DOI:** 10.1021/acs.jpca.4c03612

**Published:** 2024-08-28

**Authors:** Léon L. E. Cigrang, Graham A. Worth

**Affiliations:** Department of Chemistry, University College London, London WC1H 0AJ, United Kingdom

## Abstract

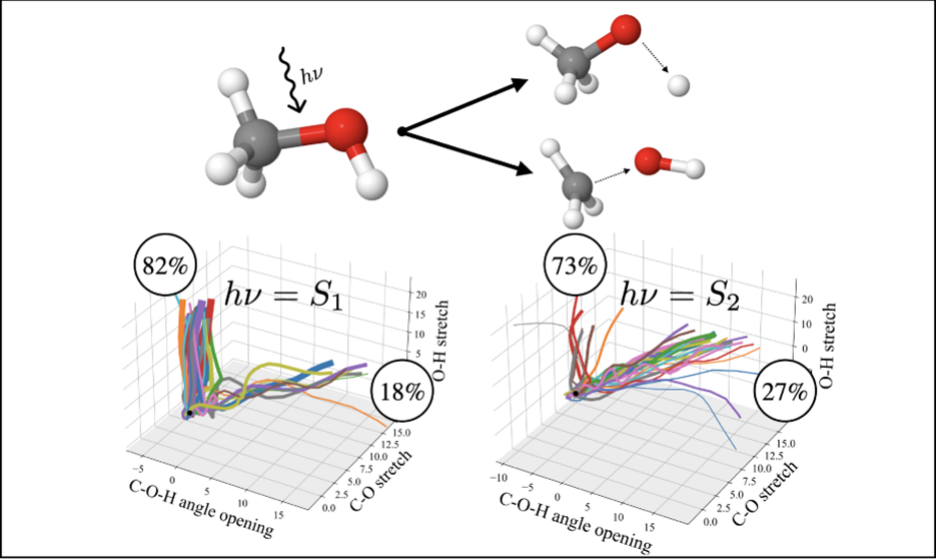

A comprehensive computational study of the gas-phase photodissociation dynamics of methanol is presented. Using a multiconfigurational active space based method (RASSCF) to obtain multidimensional potential energy surfaces (PESs) on-the-fly, direct quantum dynamics simulations were run using the variational multi-configurational Gaussian method (DD-vMCG). Different initial excitation energies were simulated to investigate the dependence of the branching ratios on the electronic state being populated. A detailed mechanistic explanation is provided for the observed differences with respect to the excitation energy. Population of the lowest lying excited state of methanol leads to rapid hydroxyl hydrogen loss as the main dissociation channel. This is rationalized by the strongly dissociative nature of the PES cut along the O–H stretching coordinate, confirmed by the broad feature in the absorption spectrum. In contrast, more energetic excitations lead mainly to C–O bond breaking. Again, analysis of the diabatic surfaces offers a clear explanation in terms of the nature of the electronic states involved and the coupling between them. The type of calculations presented, as well as the subsequent analysis of the results, should be seen as a general workflow for the modeling of photochemical reactions.

## Introduction

Small organic molecules such as methanol, carbon monoxide, and ammonia are relatively abundant in our own atmosphere and undergo a range of different reactions, most of which are photochemical in nature. Interestingly, the same small organic species have also been observed in large dust clouds near star forming regions in the interstellar medium. Spectroscopic measurements of those regions of space suggest that rich photochemistry is taking place there as well.^1^ Naturally, there have been many studies trying to understand the mechanisms and dynamics of reactions taking place in these environments. Specifically, the relatively high abundance of methanol has led to it becoming one of the central molecules being studied when investigating chemistry in space.

**Scheme 1:**
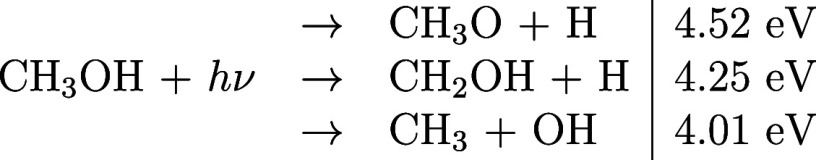
Two-Body Photodissociation Products of Methanol and Their Relative Experimental Bond Energies^2^

Upon excitation by UV radiation, methanol can photodissociate to form distinct radical species. The three photoproducts that can result from the photolysis of a single bond are summarized in Scheme 1:

The reactive species that result from this initial fragmentation now open up pathways for the formation of larger molecules such as CH_3_OCH_3_, CH_3_CH_2_OH, and (CH_2_OH)_2_.^3^ The different rates of formation for these compounds depend heavily on the branching ratio of the initial dissociation of methanol, making it important to be able to understand the dynamical behavior of photoexcited methanol molecules to ultimately gain some insight into the origin of the observed chemical complexity in space, and also to unravel the role of methanol in our own atmosphere.^4^

In previous computational work on the nature of the electronically excited states of methanol, different methods have been used to describe specific parts of the potential energy surface (PES), namely the O–H, C–O, and C–H stretching coordinates.^5−10^ This work has been used to support numerous experimental studies.^8,11−16^ By now, methanol’s UV absorption spectrum is well understood. It consists of 2 main features; a low intensity, broad band centered around 184 nm, and a finer vibronic progression at higher energy (in the region of 140–165 nm). This first feature is a clear sign of a fast dissociation and has been assigned to the transition to the first excited state which is unbound with respect to the O–H stretching coordinate, leading to rapid hydroxyl hydrogen loss. The nature of the transition corresponds to an excitation to a 3s Rydberg orbital. The higher energy end of the UV spectrum corresponds to excitations to several 3p Rydberg orbitals that lie relatively close in energy. The spectral structure in this region has been found to be related largely to the C–O stretching mode. It has been found that there is an excited state dissociative channel along this coordinate as well.

Despite the number of previous studies, there is still a lack of theoretical investigations that can quantitatively account for the branching ratios observed after UV irradiation and provide a detailed mechanistic understanding of the processes involved. This demands a method able to describe the quantum dynamics (QD) of a molecule, taking into account most, if not all, of its degrees of freedom (DoFs), in addition to the coupling between the electronic states. Computationally this poses a challenge, but the past 30 years or so have seen a large amount of effort dedicated to this problem. Different ways of solving the time-dependent Schrödinger equation (TDSE) have emerged, allowing the simulation of the excited state dynamics of molecules. The now well-known multi-configurational time-dependent Hartree (MCTDH) method^17^ for example, provides a formally exact way to expand the wave function (in the limit of convergence of the time-dependent basis set) but suffers from a major drawback; the need for a global analytical PES. This means that much effort is wasted calculating the global PES rather than only the regions that are relevant for the photochemistry.

To this end, direct dynamics methods have been developed, in which the PES is calculated *on-the-fly*, only in the regions that are explored during the time propagation of the wave function. The method employed in this work is the direct dynamics variant of the variational multi-configurational Gaussian method (DD-vMCG). Although MCTDH and vMCG have a similar variational foundation, the main difference between them is that the latter uses time-dependent Gaussian functions to expand the wave function rather than a grid-based basis set. It has been shown that the use of this Gaussian basis can reduce the scaling to less than exponential (w.r.t. system size) while nevertheless producing results that converge to the MCTDH result.^18^

The DD-vMCG method has previously been used to describe photochemical processes such as the dissociation of phenol and the excited state dynamics of formamide.^19,20^ These examples also highlight the importance of having a good description of regions of degeneracy in the PESs, i.e., the crossing of states, leading to conical intersections. These regions allow for nonradiative pathways between electronic states and are therefore relevant during the dynamics simulations. To correctly describe conical intersections, one needs to account for the coupling between the electronic and nuclear motion of molecules (so-called nonadiabatic coupling), and hence, the Born–Oppenheimer Approximation (BOA) becomes invalid.^21^ All these characteristics pose a great challenge in terms of the description of the electronic structure, as well as for QD simulations, and requires the use of advanced methods that go beyond the description of the electronic ground state at equilibrium.

The present work aims to demonstrate the general applicability of the DD-vMCG method to an important but challenging problem in computational chemistry: photodissociation. This goal is achieved by providing a detailed rationale of the mechanism by which methanol decomposes following UV excitation, and accounting for the abundances of the different resulting photoproducts.

## Theory and Methodology

### Non-Adiabatic Quantum Dynamics: The DD-vMCG Method

In general, the field of quantum dynamics aims to describe the nuclear wave function Ψ with respect to the nuclear coordinates **x** and time *t*. In combination with an expression for the Hamiltonian operator, *Ĥ*, one can then solve the TDSE, written in the usual form:
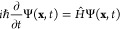
1where the Hamiltonian is just the combination of the kinetic and the potential energy terms:
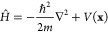
2The wave function itself is typically expanded as a product of coefficients and basis functions. In the vMCG method, the basis functions take the form of Gaussian wavepackets (GWPs), that are time-dependent, as are the expansion coefficients, *A*. This expansion leads to a linear combination of multidimensional GWPs, *G*_*i*_, as per the following ansatz:
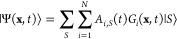
3Here,  refers to a particular electronic state *S* of the system, and *N* is the total number of GWPs. The wavepackets themselves are separable into a product of one-dimensional functions,
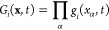
4that each take the form of a general Gaussian function:
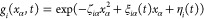
5where ζ, ξ, and η are the width, the linear and the scalar parameters, respectively, and α runs over the degrees of freedom of the system (DoFs). The vMCG formalism employs the Frozen Gaussian approximation, keeping the widths fixed and hence the time-dependence is included in the evolution of the linear and scalar parameters only.

The propagation of the wavepackets in the vMCG method obeys a set of coupled Equations of Motion (EOMs), derived from the Dirac–Frenkel variational principle,
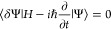
6and can be written in vector notation as follows:
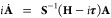
7
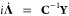
8

Equation 7 controls the coefficients *A*_*i*_, collected in **A** and Equation 8 contains the positions and momenta of the GWPs in the **Λ** vector. **H** is the Hamiltonian matrix, , and **S** and **τ** are the overlap, , and differential overlap, , matrices, respectively. All of the parameters that define the dynamics of the GWPs are collected in the **C** matrix and **Y** vector but will not be discussed here. More detailed descriptions of the derivation and discussions of the method can be found elsewhere in the literature.^22−25^

This method is well suited for the use of the *on-the-fly* calculation of the potential surfaces. This is known as Direct Dynamics, hence resulting in the DD-vMCG method. The matrix elements of eq 7 can be calculated analytically by expanding the PES using the Local Harmonic Approximation (LHA), which is a second-order Taylor expansion with respect to the centers of the GWPs, **x**_0_:
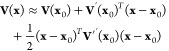
9The necessary elements to evaluate this expansion are provided by standard Quantum Chemistry programs in the form of the adiabatic potential energy (**V**), the nuclear gradients (**V′**), and the Hessian matrix, (**V″**), all evaluated at particular nuclear geometries (**x**_0_).^19^

An important point to note is that the propagation of the GWPs in the DD-vMCG method happens in the diabatic basis and hence needs diabatic PESs. This implies the need for a diabatisation scheme since the surfaces that are obtained from electronic structure methods are adiabatic. This is achieved by propagating the adiabatic-diabatic transformation matrix in a scheme known as *propagation diabatisation*.^26^ The following relationship^27^ between the transformation matrix, **D**, and the derivative coupling matrix, **F**, is used to find the new transformation matrix at a point **x** + **△x** from the initial matrix **D**(**x**):
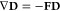
10The gauge for the propagation is then set by taking the diabatic and adiabatic states to be equivalent at the Franck–Condon (FC) point. In this way, **D** can be evaluated at each point along the PES. It should be noted though, that the relationship in eq 10 is exact in the limit of the set of infinite electronic states only, and therefore may not hold in realistic calculations, where only a subset of states is considered. However, the applicability of this diabatisation scheme in the context of the DD-vMCG method has been previously demonstrated for multistate molecular systems, and hence, its validity is assumed.^28^

### Computational Details

#### Electronic Structure

The choice of electronic structure is extremely important as it will ultimately determine the quality of the dynamics.^29^ The selected method needs to be able to describe the phenomena that one is interested in, which in this case relates to bond dissociations. This is widely recognized as a significant challenge for electronic structure methods since the molecule will be distorted far from equilibrium.^30^ The so-called multireference methods are most suitable for these types of calculations since, as opposed to most other types of methods, they do not rely on a single ground-state reference wave function.^31^ Furthermore, the method has to be able to describe excited states in order to simulate photoinduced processes.

A popular choice for this class of problems are the active space based methods. These consider every possible electronic configuration within a subset of chosen orbitals (i.e., the active space). In this work, the restricted active space self consistent field (RASSCF)^32^ method was chosen for all excited state calculations, after being benchmarked against comparable calculations (see Table 1). This method is analogous to the widely used CASSCF method, the only difference being that the active space in RASSCF is subdivided into 3 subspaces; RAS1, RAS2, and RAS3. RAS2 is the active space in which all electronic configurations are computed while RAS1 and RAS3 consist of a certain number of occupied and unoccupied orbitals, respectively. One can then specify how many electron holes will be created in RAS1 and how many electrons are allowed to be excited into RAS3. This approach significantly reduces the computational expense and has been shown to achieve similar accuracy compared to CASSCF.^33,34^ However, the accuracy of active space based methods rely heavily on the choice of orbitals included in the active space, and when it comes to this, there is no well established procedure except for a few general rules.^35^

**Table 1:** Vertical Excitation Energies of the Lowest Lying Electronic States of Methanol (in eV), Calculated Using the EOM-CCSD, CASSCF(12,12), CASPT2(12,12), and RASSCF(12,2+8+2)[1,1] Methods, Compared to Experimental Values^a^

label^b^	excitation	EOM-CCSD	CASSCF	CASPT2	CASPT2*^c^	RASSCF	exp^8^
S_1_	2p_y_(O) n 3s Ryd.	6.7014	6.3223	6.7981	6.5217	6.3343	6.7596
S_2_	2p_*y*_(O) n 3p_*x*_Ryd.	7.8981	7.4904	7.9547	7.6860	7.5056	7.7268
·	2p_*y*_(O) n 3p_*x*_Ryd.	8.4861			8.2491		8.3133
·	2p_*y*_(O) n 3p_*z*_Ryd.	8.5791			8.2637		8.3133
S_3_	σ(OH) 3s Ryd.	8.7349	8.4232	8.8367	8.6212	8.4374	/d
S_4_	σ(OH) 3p_*x*_Ryd.	9.7099	9.2984	9.7562	9.5900	9.3143	/
S_5_	2p_z_(C) 3s Ryd.		10.9836	11.3136		11.0046	

a The aug-cc-pVDZ basis set was used for all calculations.

b “*S_n_*” refers to the adiabatic singlet states at the FC point of the RASSCF results.

c Active space is changed to include all 3p Rydberg orbitals, instead of the σ^*^ orbitals.

d Not explicitly seen experimentally due to low oscillator strength.

In order to describe the photodissociation of methanol, the σ and σ^*^ orbitals of both the C–O and O–H bond were included in the active space. Furthermore, a lone pair on the oxygen atom was included along with the 2 lowest lying Rydberg states (3s and 3p_x_) that correspond to the LUMO and LUMO+1, respectively. Although these orbitals are the most important, the entire active space consisted of 12 electrons and 12 orbitals in total, subdivided to give RASSCF(12,2+8+2)[1,1] (where the notation RASSCF(*n,m*^I^+*m*^II^+*m*^III^)[*h,e*] refers to *n* active electrons in *m*^*I*^ RAS1 orbitals with a maximum of *h* holes, *m*^*II*^ RAS2 orbitals and *m*^*III*^ RAS3 orbitals with a maximum of *e* electrons).

**Figure 1:**
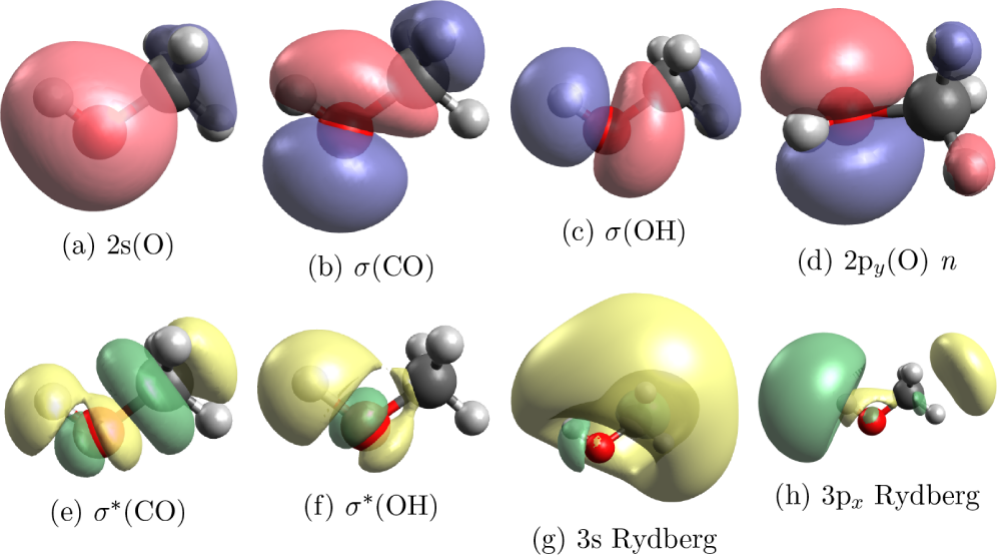
Molecular orbitals included in the RAS2 active space. (a–d) are initially occupied while (e–h) are unoccupied orbitals.

This method, as implemented in the *OpenMolcas* package,^36^ was used to evaluate the potential energy during the Direct Dynamics simulations. The computation of the nuclear gradients and nonadiabatic coupling vectors is also required but since the analytical calculation of these properties is not available for the RASSCF method, they were calculated at the CASSCF level of theory. The active space is now simply limited to the 8 orbitals within the RAS2 space, with 8 active electrons. The orbitals of this active space, calculated using the aug-cc-pVDZ basis set, are presented in Figure 1.

As in previous DD-vMCG studies^19,20,37,38^ the potential surfaces and diabatic couplings required for the DD-vMCG propagation are formed by Shepard interpolation of the RASSCF energies rotated into a diabatic picture. The diabatisation is done using the propagation diabatisation procedure that forms the adiabatic-diabatic transformation matrix by propagating the nonadiabatic coupling vectors.^22^ The Hessians are also required for the interpolation and these are calculated at the initial point (again at the CASSCF level) and then a Hessian updating scheme is used for subsequent points.^39^ Thus, despite calculating gradients and couplings at the CASSCF level, the dynamics are run on RASSCF diabatic potentials and couplings.

#### Quantum Dynamics

The QUANTICS package^40^ was used for all QD simulations as well as the subsequent analysis of the data. The data presented is the result of simulations where 33 GWPs were propagated, parametrized with a width of  and including 11 orthogonal coordinates (i.e., all but one of the normal modes). The methyl rotational mode was frozen since converting torsional motion from normal mode coordinates to Cartesian space is problematic due to the linear displacement vectors, which do not adequately describe the curved path of the rotating atoms.^41^ Initially, the wave function is constructed with all wavepackets centered at the FC point. To represent an exact vertical excitation, a momentum-space distribution of the wavepackets is used but the initial population goes solely to the wavepacket with zero initial momentum. The 11-dimensional configurational space is generated by performing multiple, sequential *on-the-fly* calculations. All the electronic structure points generated during these simulations are stored in a database, which is updated with each additional run. A Shepard interpolation between the database points is then used to obtain analytical expressions that describe the potential energy landscape, not only along the pure normal mode coordinates, but across all relevant regions explored during the simulations. When the PESs based on the database are described by a significant number of points covering the space, a final quantum dynamics simulation is run *without* updating the database further. In the present case, the final database contained 1500 quantum chemistry points. Using this data, 1-dimensional cuts can be generated, for example along the key dissociative modes, i.e., O–H and C–O stretches (see the Supporting Information for the PES cuts using the database points) to make sure that these have the correct topology.

Three separate calculations were run where the wavepackets were initially excited vertically to the first, second and third electronic states. Throughout the paper, the symbols *X̃*, *Ã*, *B̃*,... are used to denote diabatic states and S_*n*_ to denote the adiabatic states. As the dynamics run in the diabatic picture, these three calculations are excitations to *Ã*, *B̃,* and *C̃*, which at the FC point correspond to the lowest three adiabatic states: S_1_: *n* → 3s Ryd., S_2_: *n* → 3p Ryd. and S_3_:  3s Ryd.

Although methanol has three different types of bonds that could break; C–O, O–H, and C–H, only the first two are expected to occur as a result of direct photodissociation. The C–H bond has only been reported to dissociate on long (picosecond) time scales, either after a methanol cation is created,^16^ or through the O(^1^*D*) + CH_4_ reaction.^14^ These processes are outside the scope of the current work. Here, we focus on ultrafast direct dissociation reactions, caused by unbound excited state channels.

In practice, a cutoff point needs to be defined for dissociative channels, to avoid having to describe the molecule at very long bond lengths. Following an approach commonly used in grid-based methods, Complex Absorbing Potentials (CAPs) were used to limit the dissociative motion of the wavepackets. These CAPs are negative, imaginary potentials defined as follows:
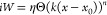
11with an order, *n*, a strength, η, and Θ being the Heaviside step function. The parameter *k* simply defines if the CAP, positioned at *x*_0_, is in the positive or negative direction along a normal mode. One important difference between CAPs in grid-based methods and the ones used here should be noted. Whereas wavepackets moving on a grid need to be absorbed smoothly by a CAP to avoid nonphysical reflections from the end-points, the Gaussian wavepackets in the DD-vMCG method are simply uncoupled from all other GWPs after crossing the dividing surface into the CAP. At this point they continue their motion as classical wavepackets until their population drops to zero, absorbed by the imaginary potential. This is necessary for two reasons, the first being that the electronic structure calculations may become unreliable at highly nonequilibrium geometries. The second is related to the integrator, which will have to reduce the time step as a dissociating fragment accelerates away from the parent species, causing the total simulation time to increase sharply.^19,20^

All CAPs were parametrized with *n* = 3, η = 0.01 and placed along the following normal modes at suitable positions so that they did not interfere with vibrational motion (positions given in brackets in units of mass-frequency weighted normal modes, as well as in Å for bond lengths, and degrees for angles): O–H stretch (20 = 2.9 Å), C–O stretch (13 = 2.7 Å), and C–O–H angle bend (15 = 151°). Note that the C–O–H angle coordinate has to be included to properly describe the C–O dissociation channel since the breaking of this bond goes along with an opening of the angle. Wavepackets reaching the CAPs causes the magnitude of the overall wave function, given by the square of the norm (), to decrease. The simulations were run until , i.e., until the molecule has fully dissociated, or until a simulation time of 100 fs was reached.

In addition to the DD-vMCG calculations, a regular vMCG calculation was also run to calculate an absorption spectrum and hence validate the electronic structure calculations. Since this method does not use the *on-the-fly* updating of the PES, a global representation of the energy landscape is required. This was obtained by constructing a model Hamiltonian from a linear vibronic coupling model.^42^ Details of the Hamiltonian and spectrum are in the Supporting Information. The model consists of analytical PESs, fitted to calculated points along the key normal modes of methanol (O–H and C–O stretching, C–O–H angle bending, and CH_3_–OH rocking). It should be noted that the fitted surfaces in this model merely approximate the actual topology of the complex energy landscape. Nevertheless, the spectrum obtained from the vMCG calculation using this Hamiltonian reproduces the main features seen in the experimental data. The broad, low intensity band is a characteristic sign of fast hydroxyl hydrogen loss and the higher energy peaks are due to C–O vibrational motion. Only a relatively small energetic shift was applied to position the spectrum on top of the experimental one. This spectrum, obtained from the same electronic structure calculations as the DD-vMCG simulations, provides an indication that the choice of electronic structure method is appropriate.

## Results and Discussion

### Electronic Structure Benchmark

A benchmark of electronic structure calculations is presented in Table 1. The values in this table correspond to vertical transition energies at the FC geometry (coordinates in the SI, obtained by optimization at Coupled Cluster Singles and Doubles (CCSD) level). In this region, at or near equilibrium, all of these methods are expected to be suitable for the calculation of excited states. The results obtained using the Equation-of-Motion CCSD (EOM-CCSD) method are useful to use as a benchmark for the other methods since it is in principle a ″black-box″ calculation, meaning that the user does not need to make any judgments about what to (not) include in the calculation, and therefore no bias is introduced. However, being a single-reference method, one might expect the accuracy to deteriorate at geometries far away from the ground state equilibrium.

The active space based methods (CASSCF, CASPT2, and RASSCF) on the other hand, are more appropriate for this system since electronic degeneracies and bond breaking can be correctly described.^31^ However, compared to the EOM-CCSD and experimental results, some low lying 3p Rydberg states are missing. This is simply due to the fact that only the 3p orbital necessary to describe the S_2_ state was included in the active space. This was done in order to keep the active space consistent with respect to distortion away from equilibrium. As the bonds of the molecule are stretched, the σ^*^ orbitals are lowered in energy and it is crucial to include these in the active space, as opposed to the 3p Rydberg orbitals corresponding to bound states, which quickly increase in energy. Since the aim of this work is to describe the dissociation of the bonds, this trade-off is deemed appropriate. To test this further, CASPT2 calculations were done using an active space that does include the other two 3p Rydberg orbitals (see CASPT2* in Table 1). However, this was not a suitable starting point for *on-the-fly* dynamics as the method was not stable when undergoing significant distortion away from the equilibrium geometry. This is because the orbitals in the active space change during the simulation, causing discontinuity in the energy. The omission of these two states in the calculations is not likely to have a large effect on the photochemical dynamics of the molecule since they are very similar in nature to the S_2_ state (i.e., bound Rydberg states), only lying slightly higher in energy.

**Figure 2:**
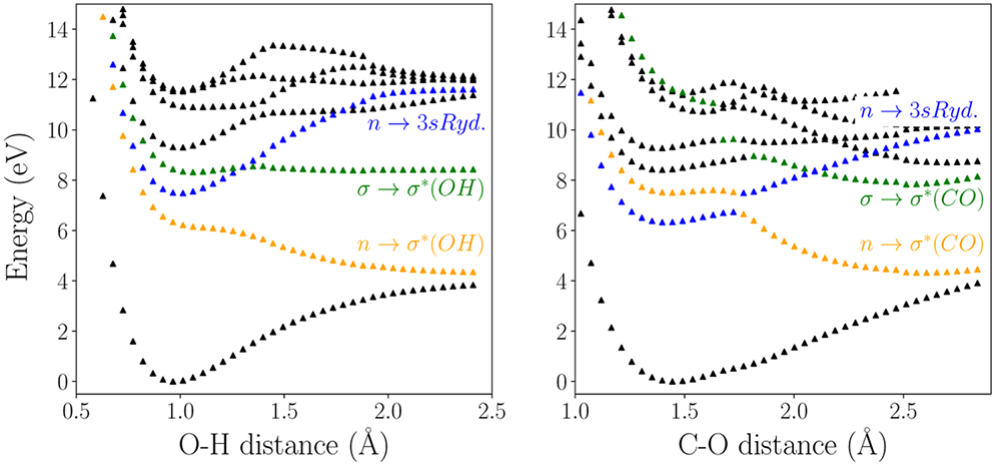
Calculated RASSCF(12,2+8+2)[1,1] points along the O–H stretching (left) and C–O stretching + bending (right) modes of methanol. The three low lying diabatic states are labeled with the character of the main tranistion at the Franck–Condon point.

The CASSCF results seem to slightly underestimate the excitation energy of the states. This can indicate that the inclusion of dynamical correlation is important. The PT2 variant of the method (CASPT2) can be used to correct for this using a second-order perturbation to the wave function. Now, the choice between using CASPT2 or RASSCF can be made based on how well the relevant parts of the potential energy landscape are described. This is difficult to determine *a priori* and therefore, both methods were used to calculate PES cuts along the two dissociative coordinates, for 8 electronic states (see Figure 2 for RASSCF results and SI for analogous CASSCF and CASPT2 plots). The CASPT2 results suffered from a large contribution of intruder states, making the calculation unstable at certain geometries, causing discontinuities. This is a known problem with methods relying on perturbation theory, especially when also using diffuse basis sets to describe Rydberg orbitals.^43^ The issue of intruder states is usually mitigated by including a level shift but this did not seem to be effective in this case.

Going beyond standard CASPT2, it is possible to use variants of the ″multistate″ formalism such as Rotated Multi-State (RMS-CASPT2) or Extended Multi-State (XMS-CASPT2), which can correct for some of the shortcomings of the PT2 approach. Some preliminary calculations were performed using these alternatives but they were ultimately abandoned because of the increased computational cost and the fact that some of the instabilities in the standard CASPT2 calculations were still present. Moving away from active space based methods, there exist other approaches that should be able to handle the multireference nature of the problem at hand. These include the Density Matrix Renormalization Group (DMRG) or Quantum Monte Carlo (QMC)-type methods. They were not considered since derivatives and couplings may not be readily available, as well as the fact that their computational cost may limit their use in these direct dynamics simulations. Additionally, there is no interface to either of these methods in Quantics, at least for now.

The electronic structure method for use in the DD-vMCG simulations was thus chosen to be RASSCF(12,2+8+2)[1,1], which was remarkably stable during the calculation of cuts through the PES along the two key modes, shown in Figure 2. It must be stressed that these cuts are not pure bond scans but are along normal mode coordinates and made to provide an easy comparison with the surfaces obtained in the direct dynamics simulations and show the available channels. The O–H stretching mode is essentially a pure bond stretching mode but the C–O stretching mode needs to be combined with the C–O–H angle bending mode in order to reveal the dissociation channels. As the OH fragment moves away from the methyl radical, the angle also opens up. Hence, the right panel of Figure 2 is actually showing the cut along the combination of the two relevant modes for C–O dissociation, i.e., stretching and bending. These cuts are in good agreement with previously calculated surfaces found in the literature.^7,8,10,12,16^ Finally, it should also be noted that the RASSCF calculations are considerably faster than the CASPT2 ones, making the QD simulations much more manageable in terms of CPU time.

### Electronic State Character

Having found a suitable electronic structure method, the character of the adiabatic states can now be determined. These are ordered energetically but do not necessarily retain their chemical character. It is therefore useful to analyze how the character changes along the adiabats in order to identify crossings between two diabatic states (which do retain their character). The state characters were determined by analyzing the configuration interaction (CI) coefficients of the electronic configurations contributing to each state. Analysis of the coefficients in a RAS calculation can be used to evaluate the multireference nature of excited states (since the CI calculation is complete within the RAS2 active space), and hence, it is deemed a suitable measure for the characterization of the chemical nature of a state.^44,45^

**Figure 3:**
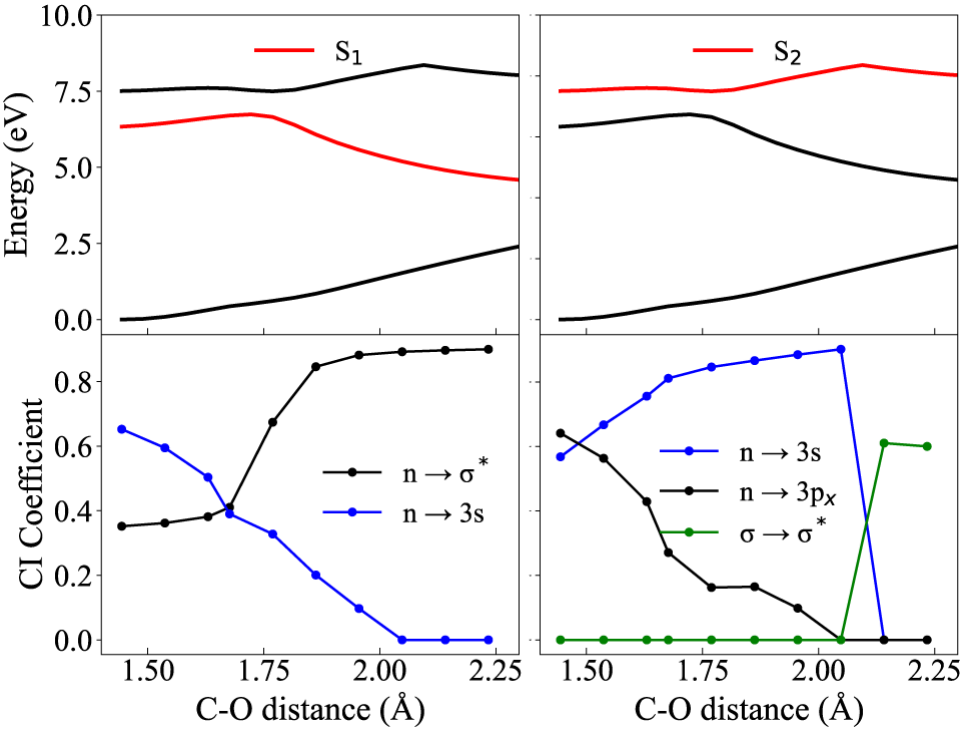
Calculated potential energies of the first 3 adiabatic states (top) (see Figure 2 for full PESs), along with CI coefficients (bottom) for the S_1_ (right) and S_2_ (left) states along the C–O stretching + bending coordinate. Calculations at the RASSCF(12,2+8+2) level of theory.

**Figure 4:**
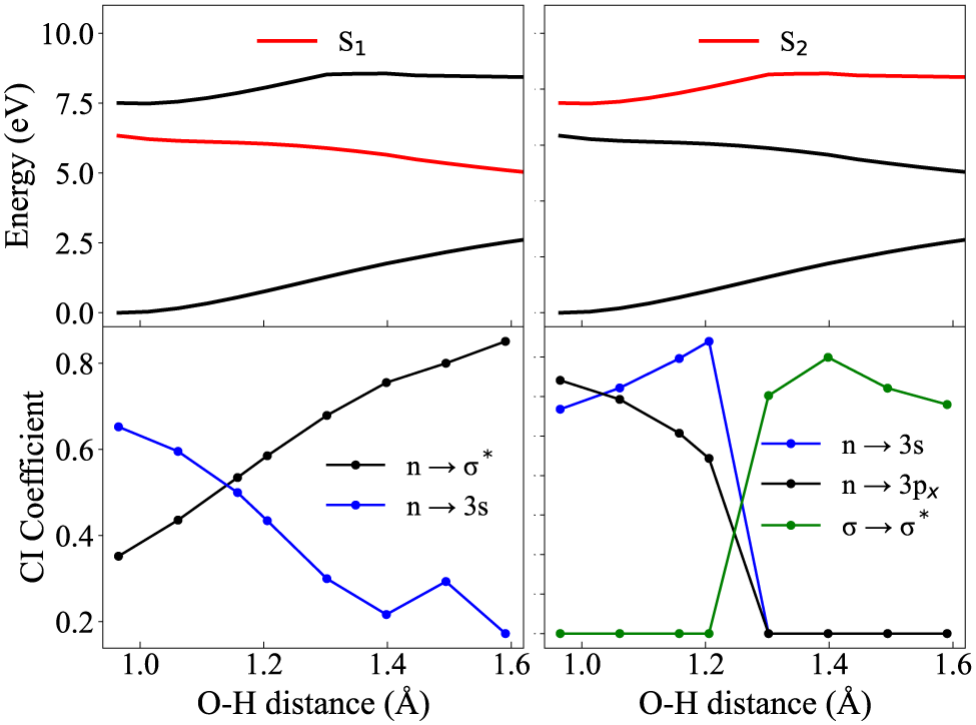
Calculated potential energies of the first 3 adiabatic states (top) (see Figure 2 for full PESs), along with CI coefficients (bottom) for the *S*_1_ (right) and *S*_2_ (left) states along the O–H stretching coordinate. Calculations at the RASSCF(12,2+8+2) level of theory.

Figures 3 and 4 show this variation in electronic configuration (given by the CI coefficients in the lower panels) of the adiabatic states (plotted in the top panels, in red, for S_1_ and S_2_ respectively). The labels of the transitions are defined based on the orbitals at the FC point which are then tracked as the geometry changes. The nature of the orbitals may also change as the bond distance increases but this analysis merely looks at the leading CAS configuration (given by the coefficients) and assigns each state with the corresponding orbital transition. The aim is to show how avoided crossings between states lead to a change in the electronic wave function of a particular state, hence emphasizing the importance of obtaining diabatic surfaces. One clearly sees, by comparing the top and bottom panels of Figures 3 and 4, how the abrupt change in gradient of the (red) adiabatic states coincides with a change in dominant electronic configuration. For both C–O and O–H dissociation, the *S*_1_ state encounters an avoided crossing with *S*_2_ which leads to antibonding character (n→σ^*^) at large bond distances. A similar trend is observed when following the *S*_2_ state, which at the FC point is characterized by an excitation into a 3p_*x*_ Rydberg orbital, but at large distances also gains antibonding character (σ→σ^*^). These two antibonding states are unbound with respect to bond stretching and hence form two (separate) excited state dissociation channels.

Transferring the above analysis into a diabatic picture, it is possible to label the surfaces in Figure 2 with their chemical character. These diabatic curves can then be compared with the PESs produced by the DD-vMCG simulations (see Supporting Information). As described in the Computational Details, 33 11-dimensional GWPs were propagated for up to 100 fs on the potential surfaces obtained *on-the-fly* (but stored in a database) using the RASSCF wave function. The initial wavepacket represented a vertical excitation of the ground-state wave function into the *Ã*, *B̃*, or *C̃* states. The diabatic cuts of these states along the O–H stretching and C–O stretching + bending modes are provided in the Supporting Information. While they are not very smooth, the main features of the static cuts of Figure 2 are reproduced. Namely, the important avoided crossings and the different dissociation pathways at different energies. The use of the propagation diabatisation scheme during the simulations allows for the determination of diabatic states, from the calculated adiabatic data. In combination with the above discussion of the electronic character of the states, the first three diabatic states can be defined, along two dissociation channels. They are summarized in Table 2.

**Table 2:** Electronic Character of the First 3 Diabatic States, along the O–H and C–O Dissociation Channels^a^

	*X̃*	*Ã*	*B̃*	C̃
O–H	g.s.	*n* → σ^*^(OH)	*n* → 3*sRyd*	σ → σ^*^(OH)
C–O	g.s.	*n* → 3*sRyd*	*n* → σ^*^(CO)	σ → σ*(Co)

a Dissociative states are underlined (g.s. = electronic ground state).

### S_1_ Excitation

First, the results of the DD-vMCG simulation are presented in which the wavepacket is initially excited to the first excited state, *Ã*.

#### Population Transfer

**Figure 5:**
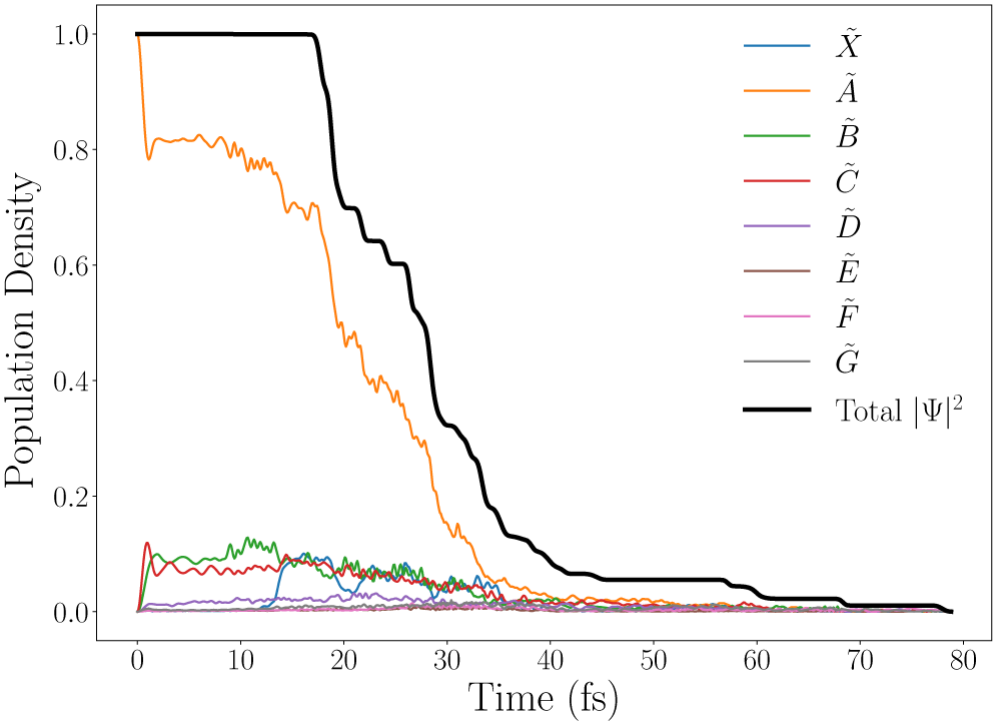
Change in the magnitude of the total wave function norm (black line), as well as the populations for each diabatic electronic state (colored lines). Initial excitation to *Ã*(S_1_).

In order to determine if dissociation has taken place, and on what time scale, the norm of the total wave function () is calculated. In a closed system, the norm (and its square) should be conserved. However, CAPs are used to absorb dissociating parts of the wavepacket which reduces the norm of the overall wave function. This can be seen in Figure 5.

This figure also shows the diabatic population density of each individual electronic state, giving an insight into which states are mostly responsible for the dissociation. During the first 20 fs, there is some internal conversion from the initially populated state (*Ã*) to the *B̃* and *C̃* states, indicating a strong nonadiabatic coupling between them, in accordance with the previously discussed state crossings. It is important to note that the very fast initial population transfer within the first few fs is not a nuclear effect, but occurs due to mixing of the initial diabatic states to form the appropriate electronic eigenstate of the system, an effect caused by the inclusion of nonadiabatic coupling terms in the diabatic Hamiltonian. After about 20 fs, the CAPs start absorbing some of the wavepacket, causing the norm to quickly drop. This clearly indicates a very rapid dissociation, mostly along the *Ã* state. Furthermore, the diabatic state population decay of the *Ã* state was used to asses the overall convergence of the dynamics simulations, by comparing the results of identical calculations with an increasing Gaussian basis (included in the SI). This comparison shows that the results are not fully converged, causing some inaccuracies in the quantities provided, but the basis set used should be reliable for the mechanistic information sought.

#### Photoproducts and Branching Ratio

Next, the photodissociation products will be determined. There are three possibilities for the initial fragmentation; CH_3_OH → CH_3_ + OH, CH_3_O + H and CH_2_OH + H. These can be related to the wavepackets by calculating the expectation value of the center coordinates of each GWP, allowing for an analysis of the configurational space explored by the wavepackets in terms of trajectories. Hence, each individual GWP can be assigned to one or none of the three channels. To provide product ratios, this trajectory analysis must be combined with another property to provide a weight for each GWP, the Gross Gaussian Population (GGP).^46^ The GGP is derived from the density of the total wave function given by the overlaps, **S**, of the basis functions and the coefficients, **A**:
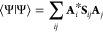
12by evenly dividing the overlaps between the Gaussian functions and taking only the real-valued part. The GGP is then calculated as follows for the *i*^*th*^ GWP at time *t*:
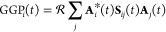
13This measure can thus be interpreted as the contribution of an individual GWP to the total wave function.

**Figure 6:**
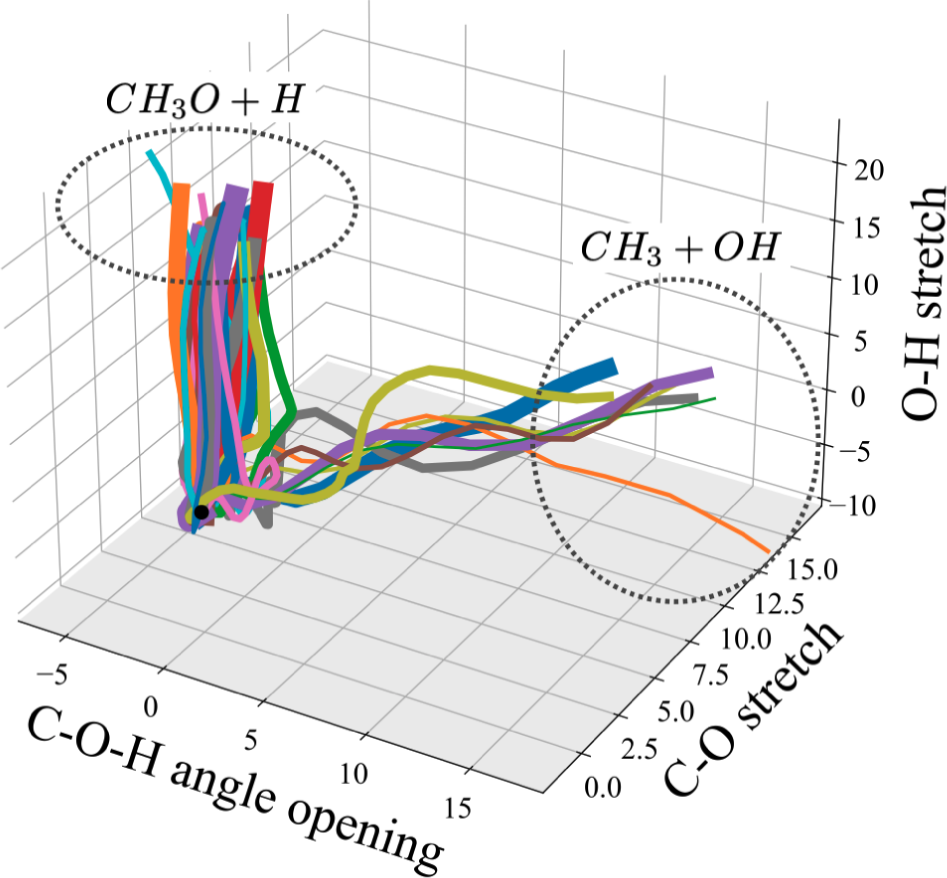
Expectation values of the centers of the individual GWPs and the resulting photoproducts after excitation to the *Ã* (S_1_) state. Line widths are proportional to the GGP associated with a trajectory and the dashed circles indicate groups of trajectories leading to the same photoproduct. Coordinates are given in units of mass-frequency weighted normal modes and correspond to the calculated modes rather than pure stretching and bending motions (see Supporting Information for mode description).

In this case, the trajectories followed by the 33 GWPs all resulted in either the breaking of the O–H bond or the C–O bond. No C–H bond breaking was observed. This is visually represented in Figure 6 by plotting the trajectories of each wavepacket in the space spanned by the O–H and C–O bond stretching coordinates, as well as the C–O–H angle bending coordinate. It is important to emphasize here that these trajectories are not individual classical trajectories, but rather the expectation values of the coupled quantum wavepackets. The thickness of the lines in Figure 6 are proportional to the GGP, i.e., thicker lines carry more weight in the overall wave function.

The trajectories reinforce the idea that O–H dissociation is a direct process, while the C–O bond also requires the C–O–H angle to open up and more space is covered in the process. By summing up the GGPs of the wavepackets leading to a certain channel, a quantitative branching ratio is obtained: CH_3_O + H: CH_3_ + OH = 0.82:0.18. The lower percentage of C–O dissociation can easily be rationalized by the energetic barrier on the *S*_1_ surface along this coordinate ( 0.45 eV, compared to the experimental estimate of 0.58 eV.^47^ These results are in excellent agreement with the experimental branching ratio of CH_3_O + H: CH_3_ + OH = 0.86: 0.14 ± 0.10, determined for photoexcited methanol at 193 nm (i.e., *S*_1_ excitation).^48^ It is noted at this point that the agreement with experiment may be fortuitous since the calculations presented are not converged. Importantly though, it demonstrates that the method is able to provide accurate results provided the underlying electronic structure is valid, and a convergence criteria is met. It hence shows promise for future applications to other multidimensional molecular systems.

#### State-Specific Dissociation

As a final step of the analysis, the relative contribution of each individual electronic state to the total dissociation will be discussed. This is calculated by measuring the amount of density that reaches the dissociation channels for each state separately, which is obtained by the flux going into the CAPs. This type of analysis is useful to determine not only which photoproducts are formed but also what electronic character they correspond to as there may be multiple dissociation pathways leading to the same products but in different states. When initially exciting the *Ã* state (dissociative with respect to the O–H bond) it is of course expected that most of the dissociation will be through this state. Indeed, this is confirmed by the data presented in Table 3 for the major O–H dissociation channel, although a significant fraction dissociates after crossing to the ground (*X̃*) state. It should be noted that the contribution of the other states, although small, is not negligible. These higher lying dissociation channels will become more important as the excitation energy increases, as will be shown later. The minor C–O channel is seen to dissociate with more evenly spread distribution over the 5 states.

**Table 3:** Contribution of Individual Electronic States to the Total Dissociation after Excitation to the *S*_1_State

channel	branching ratio^a^	*X̃*	*Ã*	*B̃*	*C̃*	*D̃*
O–H	0.89	0.17	0.57	0.07	0.07	0.02
C–O	0.11	0.01	0.04	0.03	0.02	0.02

a Ratio is calculated based on the sum of the state-specific density going into each channel.

A second useful aspect of this state-specific analysis is that it is possible to obtain a branching ratio between the two channels by simply summing up the contribution of each state to each individual channel. This ratio is also shown in Table 3 and matches the ratio determined from the GGP analysis. These two methods for calculating the ratios are based on entirely different data but give the same answer, hence working as a validation for each other (in addition to the agreement with experimental data).

### S_2_ and S_3_ Excitation

To investigate the effect of excitation into the manifold of higher lying electronic states, DD-vMCG simulations were run where the second and third excited states were initially populated. The results are analyzed in the same way as the *Ã* calculation above.

#### Population Transfer

**Figure 7:**
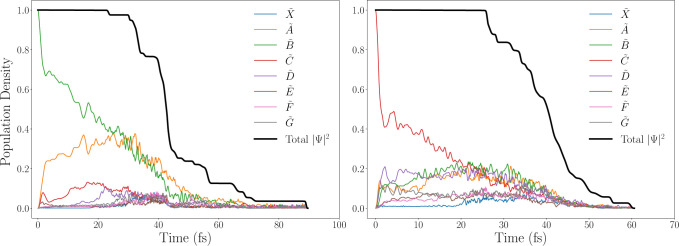
Change in the magnitude of the total wave function (black line), as well as the change in diabatic population density for each electronic state (colored lines). Initial excitation to *B̃* (S_2_) (left) and *C̃* (S_3_) (right).

We start with the diabatic state populations presented in Figure 7. Excitation to *B̃* corresponds with the population of a bound state along the O–H coordinate but a dissociative state with respect to C–O bond stretching. After an initial period of internal conversion, a rapid decrease of the total density is again observed, leading to an essentially complete dissociation after about 90 fs. Initially populating *C̃* leads to a longer period of internal conversion, after which the wavepackets start to be absorbed by the CAPs and the molecule dissociates in 60 fs.

In summary, although there are quantitative differences, the excited state population transfer of methanol can be characterized in the same way regardless of which state is initially excited. The fairly large coupling between the first 4 excited states, combined with the fact that there are multiple regions of degeneracy between the states, leads to rapid internal conversion until the wavepackets reach a dissociative state, after which they will follow the gradient of this state and are ultimately absorbed by a CAP. However, more energetic initial excitations will significantly affect *which* dissociative states are more accessible, and hence which photoproducts are more readily formed.

#### Photoproducts and Branching Ratio

**Figure 8:**
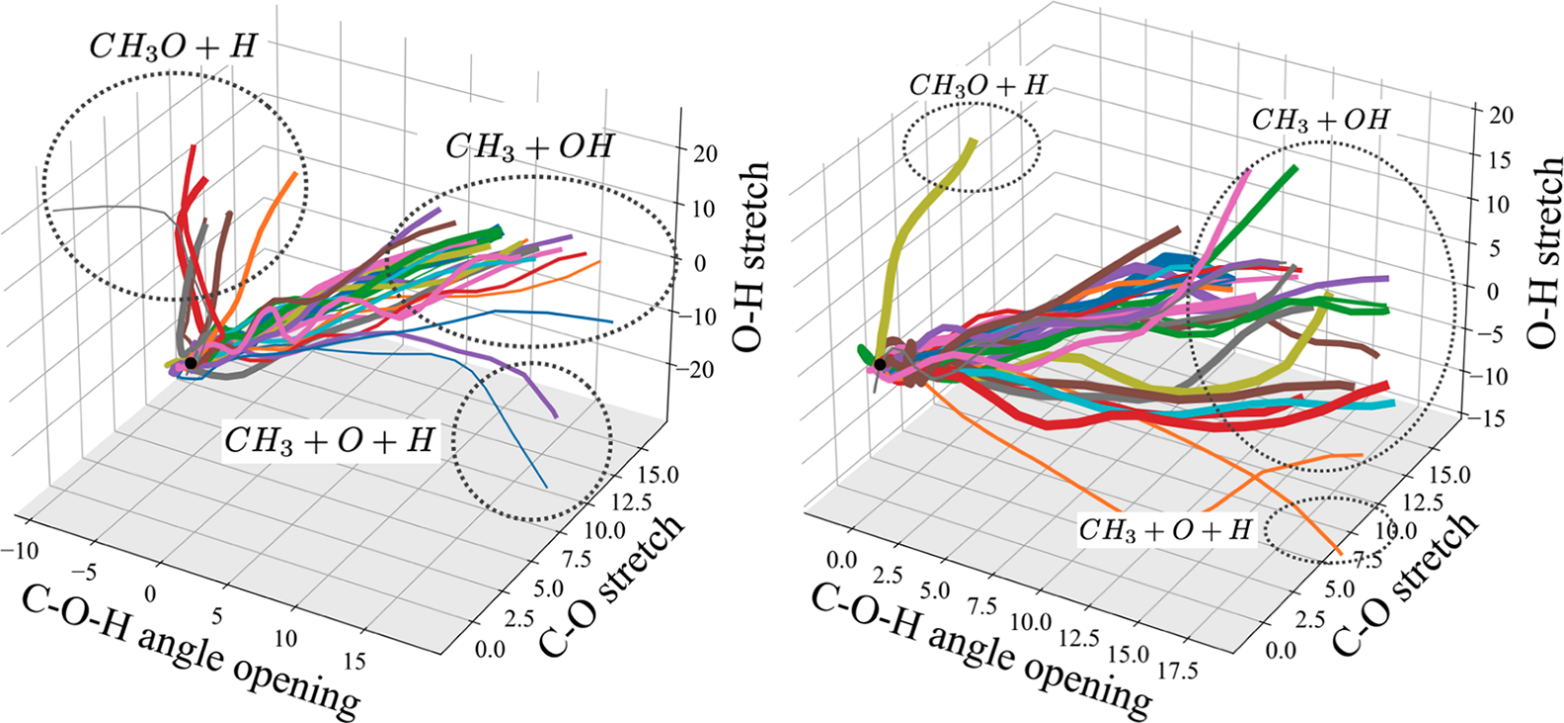
Expectation values of the centers of the individual GWPs and the resulting photoproducts for excitations to *B̃* (*S*_2_) (left) and *C̃* (*S*_3_) (right). Line widths are proportional to the GGP and the dashed circles indicate groups of trajectories leading to the same photoproduct. Coordinates are given in units of mass-frequency weighted normal modes and correspond to the calculated modes rather than pure stretching and bending motions (see Supporting Information for mode description).

The products were again determined by analyzing the expectation values of the trajectories of the basis set wavepackets (Figure 8). Comparing these results with the trajectories resulting from initial *Ã* population (Figure 6), the picture has changed dramatically. The majority of wavepackets are now driven toward the dissociation of the C–O bond, whereas only a few result in O–H scission. To quantify this, the branching ratios were again calculated by summing up the GGPs and the following results are obtained; *B̃*: CH_3_O + H: CH_3_ + OH = 0.27:0.73, and *C̃*: CH_3_O + H: CH_3_ + OH = 0.05:0.95. A three-body dissociation channel is also open. Around 5% of the wave function density leads to both C–O and O–H cleavage, resulting in a CH_3_, an O(^3^*P*), and a H radical fragment, formed after *B̃* excitation. This percentage decreases to about 2% when *C̃* is initially excited. Experimentally, a similar channel is also observed but is assigned to a two-step process whereby the methoxy radical dissociates *after* the O–H bond has been broken.^47^ In the simulations presented here, the three fragments seem to form simultaneously. These two results are difficult to compare directly since the experiment was done using a multiphoton ionization technique (REMPI) and the simulations were performed with a single initial excitation.

Overall, excitations into the manifold of higher lying excited states cause the yield of O–H dissociation to drop massively and the photochemistry becomes almost entirely dominated by the breaking of the C–O bond. This result is rationalized by comparing the PESs of both channels (Figure 2). There is an energetic barrier with respect to C–O dissociation on the first excited state and this barrier is more easily overcome by a more energetic initial excitation to *S*_2_ or *S*_3_. Additionally, the nonadiabatic coupling between the dissociative state and the state above is very strong, especially along the C–O stretching mode (confirmed by the sharp avoided crossing), which facilitates transfer to the dissociative states.

#### State-Specific Dissociation

Finally, an analysis of the state-specific contributions to the two major dissociation channels is carried out and summarized in Table 4. There is once again fairly good agreement with the ratio obtained from the GGP analysis, however the differences are now larger compared to the results obtained for *S*_1_ excitation. Likely, the reason for this is related to the fact that the density going into each state (for each channel) is calculated as a sum over all wavepackets, whereas the GGP analysis is specific with respect to which wavepackets are included. Hence, the contribution of wavepackets that do not lead to either of the two channels (such as those GWPs contributing to the three-body dissociation) can still contribute slightly to the state-specific density, but will not affect the GGP analysis. Aside from causing quantitative discrepancies in the branching ratio, this fact does not change the overall picture.

**Table 4:** Contribution of Individual Electronic States to the Total Dissociation after Excitation to the S_2_ and S_3_ States

	channel	branching ratio^a^	*X̃*	*Ã*	*B̃*	*C̃*	*D̃*
S_2_	O–H	0.18	0.04	0.10	0.02	0.02	0.01
	C–O	0.82	0.08	0.23	0.29	0.10	0.13
S_3_	O–H	0.08	0.01	0.03	0.01	0.03	0.01
	C–O	0.92	0.13	0.23	0.22	0.15	0.19

a Ratio is calculated based on the sum of the state-specific density going into each channel.

Some insight can now be gained by comparing Tables 3 and 4. When the first electronic state of methanol is excited, the result will mainly be loss of the hydroxyl hydrogen due to the repulsive PES of this state along the O–H stretch coordinate. Nearly 60% of this loss happens through direct dissociation on the repulsive Ã state. In contrast, the higher lying electronic states play a rather significant role in the dissociation of the C–O bond. The contribution of these higher states increases as the initial excitation energy goes up and this helps to explain why the branching ratios look so different between S_1_ and S_2/3_ excitation. When exciting above S_1_, the small energetic barrier along the C–O bond breaking channel is overcome and the strong coupling between the higher lying electronic states along this channel results is a strong preference of the breaking of this bond, rather than the O–H bond.

## Conclusion

With the aim of gaining a mechanistic understanding of photochemical reactions, this work exemplifies how nonadiabatic QD simulations can model, in great detail, the photodissociation reactions of small molecules in the gas-phase. The present work focuses on the dissociation of methanol but the methodology should also be seen as a flexible and general approach to study the photochemical behavior of any molecular system, provided it is small enough to achieve feasible simulation times. In terms of accuracy, the results from *on-the-fly* simulations require suitable electronic structure calculations to describe the multidimensional and multistate PESs, nuclear gradients and Hessians, as well as nonadiabatic couplings between electronic states. The accuracy of these calculations ultimately determine how well the QD simulations perform, and this part is often the main bottleneck in the entire procedure.^29^ After careful benchmarking of the vertical excitation energies, the multireference RASSCF method proved to be suitable for the description of the states of interest, and is stable along all normal mode coordinates.

The DD-vMCG method implemented in quantics was used to run QD simulations, starting from an initial excitation of the wavepackets to the lowest lying excited state (S_1_). Additionally, two more calculations were done to investigate the effect of more energetic excitations (now initially populating the S_2_ and S_3_ states, separately). From these simulations, two different dissociation pathways (CH_3_OH → CH_3_ + OH and CH_3_O + H) were identified and branching ratios were determined. Although the accuracy of these quantities can be questioned since the simulations were not converged, it is demonstrated that extracting useful properties from the final calculations is straightforward and, in principle, can converge toward quantitative accuracy. The initial excitation energy is observed to have a large effect on the relative ratios. Upon S_1_ excitation, 82% of the molecular wave function leads to O–H bond breaking whereas this ratio is completely reversed when exciting into the higher lying manifold of states (i.e., S_2_/S_3_). This observation is rationalized by analysis of the diabatic PESs. The Ã state is strongly dissociative along the O–H stretching coordinate (having mainly *n* → σ^*^ character) whereas it is a bound state with respect to C–O bond breaking (*n* → 3*s* character). Higher excitations however, easily overcome the small barrier toward C–O dissociation as well as accessing multiple dissociative states along the C–O coordinate, causing the dramatic change in branching ratio. It is easy to imagine how the branching ratio of a molecule impacts the chemistry that takes place after the initial fragmentation (i.e., the ultimate photoproducts that are formed). This fact makes it important to be able to accurately simulate the photodissociation of molecules that play a role in the complex chemistry of our atmosphere or in certain regions of the interstellar medium for example.

In this context, the present work is planned to be extended by studying the effects of the environment on the chemistry of a photoexcited molecule. For example, the presence of water clusters in the atmosphere is known to influence the chemistry of many species that are present and hence the complex network of chemical reactions is affected.^49,50^ Additionally, a lot of astrochemical reactions are thought to occur on the surface of small icy particles present in interstellar gas clouds.^1,3^ To study how reactions are affected by a specific environment, a novel methodology is being developed by the authors of this work, and collaborators. The aim is to explicitly model a molecular environment around electronically excited species and perform DD-vMCG calculations similar to the ones presented here. The methodology relies on certain classical approximations to reduce the overall cost of the calculations, which have been validated in recent work.^51^

## Data Availability

Data from the calculations in the form of QUANTICS input and output files can also be found at DOI: 10.5522/04/25913125.

## Supporting Information Available

This contains the optimized methanol geometry and normal modes, the CASSCF and CASPT2 PES cuts along the dissociative modes, the absorption spectrum calculated using MCTDH, a convergence study of the DD-vMCG basis set, and cuts through the potential surfaces from the direct dynamics simulations (PDF)
