# Supplementary material for: Modeling Photodissociation: Quantum Dynamics Simulations of Methanol
Source: J Phys Chem A. 2024 Aug 28;128(36):7546–57. doi: 10.1021/acs.jpca.4c03612 (PMC11403662; doi:10.1021/acs.jpca.4c03612)
Supplement: Supplementary file 1 — jp4c03612_si_001.pdf [file jp4c03612_si_001.pdf]

Supporting Information for: Modelling Photodissociation: Quantum Dynamics Simulations of Methanol

Léon L. E. Cigrang[1] and Graham A. Worth[1]

[1]Department of Chemistry, University College London, London WC1H 0AJ, United Kingdom

August 7, 2024

Contents

S1 Methanol Geometry & Normal Modes **S2**

S2 CASSCF and CASPT2 results **S4**

S3 Absorption Spectrum **S5**

S4 Direct Dynamics convergence **S7**

S5 Direct Dynamics PESs **S8**

S1 Methanol Geometry & Normal Modes

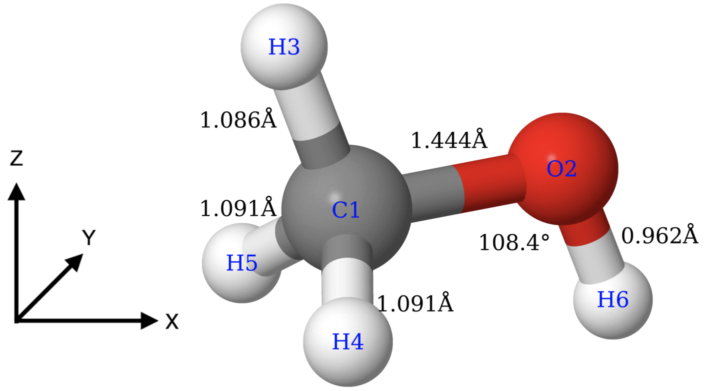

Figure S1: Geometry of methanol in C_s symmetry.

Table S1: Cartesian coordinates (in Å) for methanol used in this work, obtained after optimisation at CCSD/aug-cc-pVDZ level of theory.

**Atom**	**X**	**Y**	**Z**
C1	-0.67980632	-0.02010217	0.00000040
O2	0.75677758	0.12639138	0.00000008
H3	-1.08527514	0.98693443	-0.00000046
H4	-1.02289486	-0.54602403	0.89222483
H5	-1.02289427	-0.54602363	-0.89222447
H6	1.15320100	-0.75360198	-0.00000038

Table S2: Frequencies (in cm^{-1}) of the 12 normal modes of Methanol (CCSD/aug-cc-pVDZ)

**Mode**	**Symm.**	**Frequency**	**Description**
v_1	A''	289.0272	Torsion
v_2	A'	1026.9026	C-O stretch
v_3	A'	1045.9601	CH_3 + OH6 rocking
v_4	A''	1131.3388	H4/5-C-O angle bend
v_5	A'	1372.1551	C-O-H6 angle bend
v_6	A'	1437.1821	CH_3 symm. bend
v_7	A''	1474.1371	H3 rocking
v_8	A'	1487.4185	H4-C-H5 scissoring
v_9	A'	3154.5375	CH_3 symm. stretch
v_{10}	A''	3230.1449	C-H4/5 antisymm. stretch
v_{11}	A'	3287.6298	C-H3 stretch
v_{12}	A'	3838.8837	O-H6 stretch

S2 CASSCF and CASPT2 results

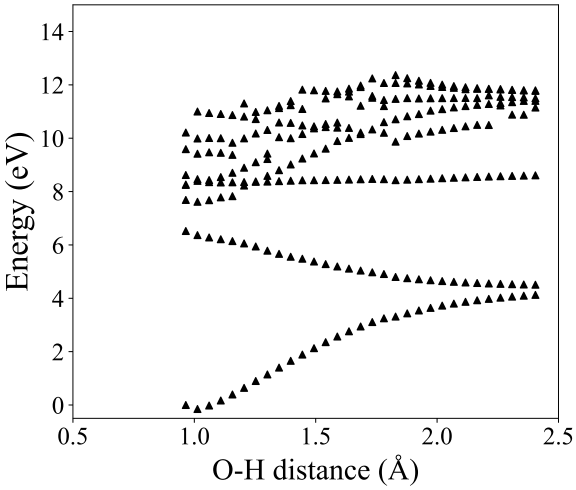

Figure S2: Calculated CASSCF(12,12) points along the O-H stretching mode of Methanol.

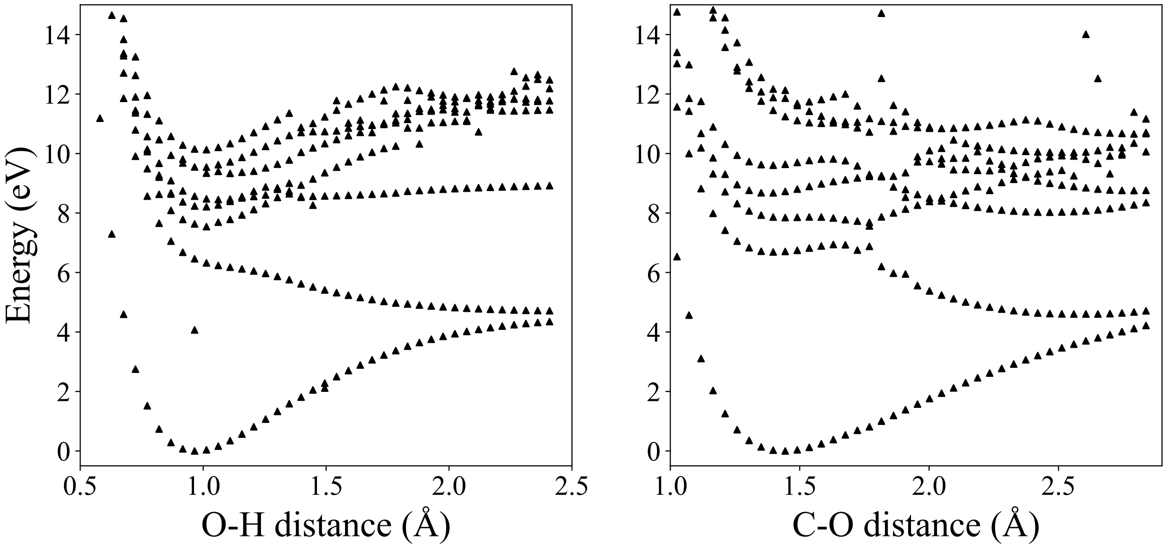

Figure S3: Calculated CASPT2(12,12) points along the O-H stretching (left) and C-O stretching + beinding (right) modes of Methanol.

S3 Absorption Spectrum

The spectrum presented here is the result of a vMCG calculation using a linear vibronic coupling (LVC) Hamiltonian, parameterised in 4 dimensions. This parameterisation is possible by representing the Hamiltonian as a Taylor series, expressed in normal mode coordinates. At the equilibrium geometry, around which the Taylor series is expanded, the diabatic and adiabatic representations of the potentials is equivalent and hence the following separation is possible:

$$\mathbf{H} = \mathbf{H}^0 + \mathbf{W}^0 + \mathbf{W}^1 + ... \tag{1}$$

where $\mathbf{H}^0$ and $\mathbf{W}^0$ are harmonic oscillators, representing the ground and excited electronic states, respectively. The oscillator is defined based on the frequencies of the normal modes of the molecule, ω_α as follows:

$$\mathbf{H}^0 = \sum_\alpha \frac{\omega_\alpha}{2} \left(\frac{\partial^2}{\partial x_\alpha^2} + x_\alpha^2 \right) \tag{2}$$

and $\mathbf{W}^0$ is simply shifted up by the excitation energy. The gradients and couplings are then provided by the on- and off-diagonal elements of the first order $\mathbf{W}$ matrix, respectively. These are commonly expressed as κ parameters for each state i, and λ parameters between each pair of states i and j:

$$\mathbf{W}_{ii}^1 = \sum_\alpha \kappa_\alpha^i x_\alpha \tag{3}$$

$$\mathbf{W}_{ij}^1 = \sum_\alpha \lambda_\alpha^{i,j} x_\alpha ; i \neq j \tag{4}$$

The wavefunction is constructed from this LVC model and is propagated using the vMCG EoMs with 256 GWPs. Finally, the spectrum is calculated from the Fourier transform of the autocorrelation function:

$$I(\omega) \propto \omega \int_{-\infty}^{+\infty} \mathrm{d}t \langle \Psi(0) | \Psi(t) \rangle e^{i\omega t} \tag{5}$$

The parameters for the LVC model are provided in the form of a Quantics operator file, along with the input files for the calculation, as part of the associated datasets to be found at DOI 10.5522/04/25913125

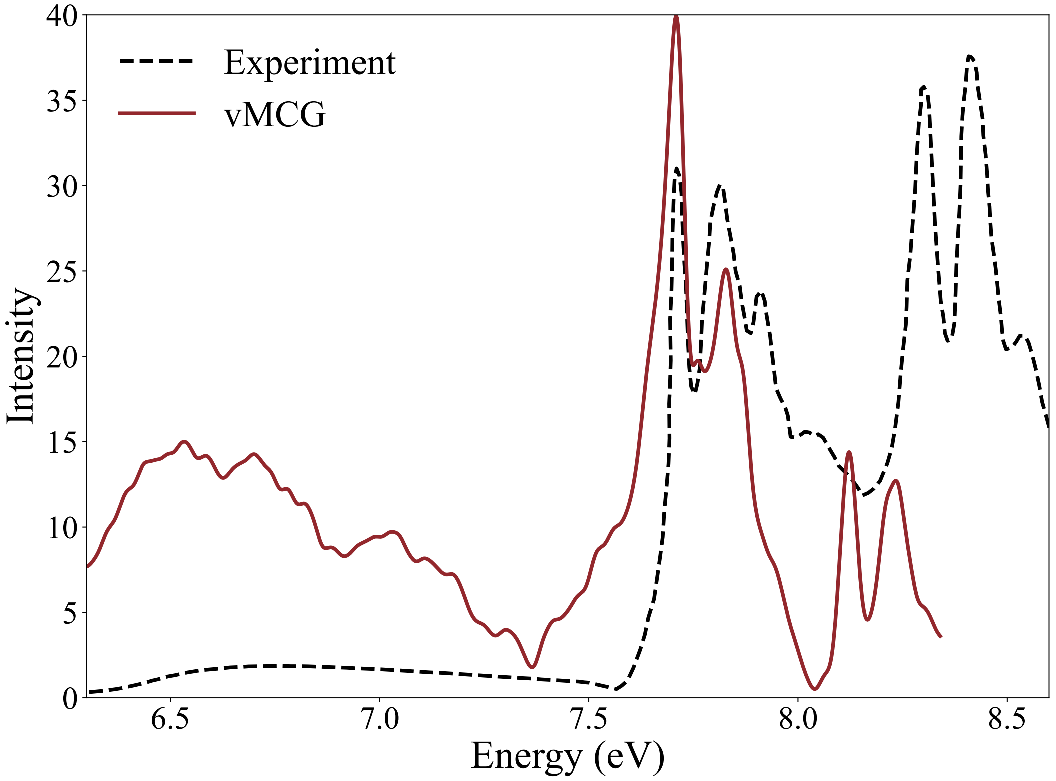

Figure S4: Calculated absorption spectrum of methanol obtained from a vMCG calculation, employing a reduced dimensionality linear vibronic coupling Hamiltonian. A horizontal shift of -0.36 eV is applied to the calculated spectrum to correspond with the experimental one.

S4 Direct Dynamics convergence

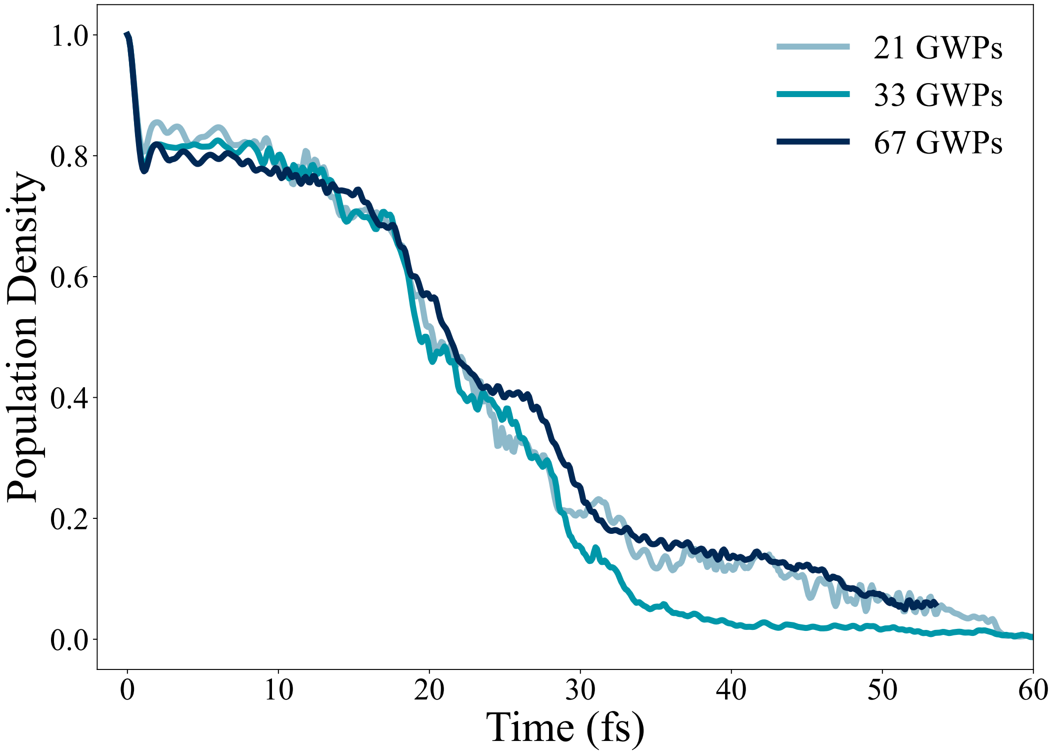

Figure S5: Comparison of the diabatic state population of the $\tilde{A}$ state of methanol, calculated using exactly the same parameters but with an increasing number of GWPs in the basis.

S5 Direct Dynamics PESs

To show the correct calculation of the relevant surfaces generated during the DD-vMCG simulations, the diabatic and adiabatic surfaces are plotted in Fig. S6. These surfaces are generated from just over 1,500 quantum chemistry calculations, stored in a database (to be found at DOI 10.5522/04/25913125). Cuts along pure normal modes are obtained by setting all other coordinates to zero, apart from the modes of interest. Apart from some ”unnatural” kinks, the surfaces adequately describe the regions that are important for dissociation, as well as the other normal modes of methanol. The diabatic surfaces clearly show the crossing between the states, leading to the important conical intersections that play a role in determining the branching ratios.

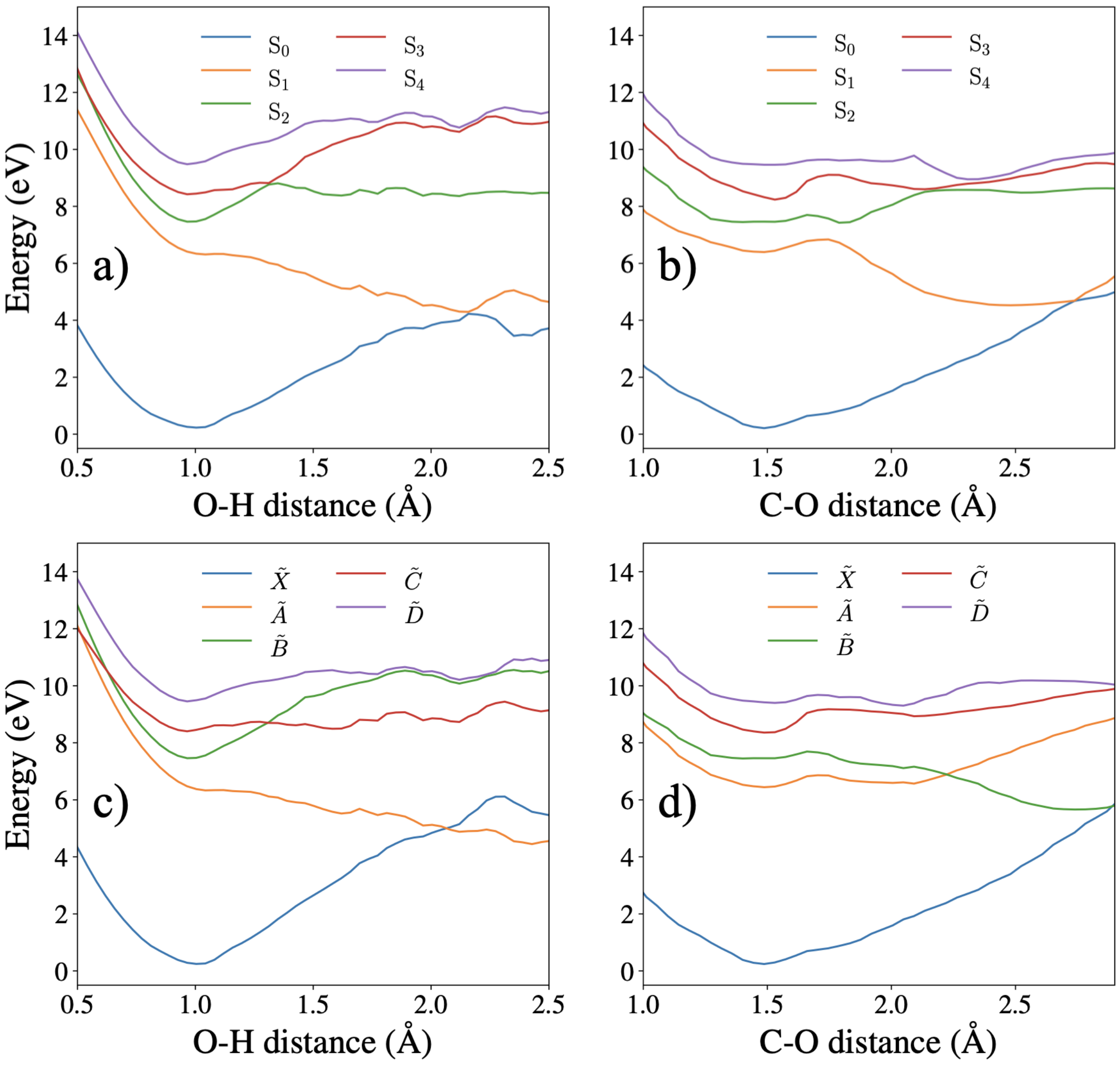

Figure S6: Cuts of the PES constructed from the database points generated during DD-vMCG simulations. Adiabatic (a and b) and diabatic (c and d) surfaces for the O-H stretching and C-O stretching + bending modes. All other modes are at zero. Note: Only the lowest 5 electronic states are shown, however a total of 8 states is calculated and included in the dynamics.
